# Lichtenstein Repair and Intersurgeon Variations: A Textbook Review and Multicenter Surgeon Survey

**DOI:** 10.3390/medicina62010079

**Published:** 2025-12-30

**Authors:** Jurij Gorjanc, David C. Chen, Andrew Kingsnorth, Reinhard Mittermair

**Affiliations:** 1 Department of General and Abdominal Surgery, Klinikum Klagenfurt am Wörthersee, 9020 Klagenfurt, Austria; reinhard.mittermair@kabeg.at; 2 Department of Surgery, David Geffen School of Medicine at UCLA, 10833 Le Conte Avenue, Los Angeles, CA 90095, USA; dcchen@mednet.ucla.edu; 3 Lichtenstein Amid Hernia Clinic at UCLA, 1304 15th Street, Suite 102, Santa Monica, CA 90404, USA; 4 Derriford Hospital, Level 7, Plymouth PL6 8DH, UK; kingsnorthandrewn@gmail.com

**Keywords:** Lichtenstein repair, inguinal hernia, hernia surgery standardization, surgical technique variations, chronic pain, hernia recurrence

## Abstract

*Background and Objectives*: A surgical method is rarely very effective and simple to perform. A Lichtenstein Repair (LR) is one such exception. Because of the very high incidence of inguinal hernia, LR has become the global gold standard in inguinal hernia repair—not only due to its relative simplicity and reproducibility but also because it can be performed under local anesthesia. These attributes facilitated its worldwide adoption, including in underdeveloped and resource limited settings. Today, many variations are performed under the common name “Lichtenstein Repair”. The extent to which these modifications influence outcomes—particularly recurrence and chronic pain—remains unclear. *Materials and Methods*: To evaluate reasons for variation in the LR technique, a literature review of seven major surgery textbooks was performed. In addition, a questionnaire comprising 17 questions addressing the key steps of the LR was sent to 90 surgeons across 19 different hospitals in Austria (6) and Slovenia (13). The questionnaire focused on core principles described by Lichtenstein and later refined by his successors. The overall response rate was 78%. *Results*: Descriptions of the LR in major hernia textbooks vary substantially, partly due to the evolution of the technique over time and partly because any subaponeurotic anterior-canal mesh repair is often labeled as “Lichtenstein”. Survey responses demonstrated considerable variation and lack of standardization or uniformity in several critical steps of the LR. More than 50% of respondents reported using pre-formed meshes that they excessively trim, limiting adequate coverage of the inguinal region. Furthermore, routine patient follow-up is lacking in the majority of cases. *Conclusions*: The contemporary umbrella term “Lichtenstein Repair” encompasses many different anterior mesh techniques. While some surgeon-specific preferences may not compromise integrity, strict adherence to the evidence-based key principles of the original repair remains essential to minimize recurrences and chronic inguinal pain. Standardization with meticulous adherence to the key principles of the LR is critical to ensure the data submitted into registries, RCTs, and meta-analyses are accurate, comparable, and meaningful.

## 1. Introduction

The lifetime incidence of groin hernia is 27–43% in men and 3–6% in women, making groin hernia repair one of the most common general surgical operations on the globe [1,2]. Since the introduction of mesh for open groin hernia repair, tension-free techniques have predominated [3]. Lichtenstein Repair (LR) is considered a minor surgical procedure and is often the trainee’s first approach to the art of surgery. Based on current evidence regarding recurrence and chronic pain, LR remains one of the most effective techniques available [4]. Irving L. Lichtenstein first described LR 37 years ago [5,6]. Its superiority among open tension-free techniques has been demonstrated in many RCTs, meta-analyses, and registries. It is referred to as the gold standard in open tension-free inguinal hernia repair worldwide due to its widespread adoption and reproducibility [6,7]. LR gained global acceptance due to its efficacy and relative simplicity. Additionally, the ability to perform the operation under local anesthesia in the ambulatory surgery setting was a significant advancement at that time [8,9,10]. With LR, surgeons found a reliable technique to address the entire spectrum of inguinal hernias globally, including in developing countries, which face the dual challenge of material constraints and large, neglected scrotal hernias [11,12]. Many surgical societies, including the American College of Surgeons, consider LR the gold standard for open inguinal hernia repair worldwide [3,6].

Despite its widespread use, key steps of LR are not consistently followed. Even amongst hernia experts, significant variation and personal modifications exist. A variety of different procedures today are performed under the common name “Lichtenstein Repair”. The exact reasons for intersurgeon variations are unclear and diverse. Many surgeons perform LR the way they were taught by textbooks or teachers and mentors, who themselves used modified or non-standard techniques. Some transitioned from tissue repairs or various open subaponeurotic mesh techniques and incorporated elements of those operations into what they still label as “Lichtenstein”. These modifications—often lacking follow-up data or evidence—are still reported as LR in registries and comparative studies [13]. However, it is not known which steps can be modified without risk for a higher recurrence rate or chronic pain [14].

Lichtenstein initially described anterior mesh repair in 1984, with further refinement over the next 4 years; LR was therefore “born” in 1988 [6]. The original method included field block anesthesia, stripping off the cremasteric muscle similar to the principles derived from prior tissue repairs, dissection of the “lesser cord” (genital branch of the genitofemoral nerve—GbGFN), sac ligation, and use of a 10 × 5 cm mesh prosthesis primarily for direct hernias, with juxtaposition of the mesh to the pubic area [5]. His protégé and successor at the Lichtenstein Institute, Parviz Amid, refined the technique—now known as Amid-modified Lichtenstein Repair—by eliminating the cremasteric resection and lesser cord dissection, which place the cord structures and GbGFN at risk of scarring to the adjacent mesh. Amid introduced local infiltrative anesthesia, lifting of the cord by 2 cm over the symphysis in the avascular plane, routine identification and preservation of the three inguinal nerves (ilioinguinal-IIN, iliohypogatric-IHN, and GbGFN), inversion of the hernia sac, preparation of the inguinal floor, and use of a larger mesh in order to extend the coverage 2–3 cm above Hesselbach’s triangle to achieve adequate overlap and most importantly 2 cm distally over the pubic tubercle [6]. He formed asymmetrical mesh tails (2/3 medially and 1/3 laterally), suturing the mesh to the inguinal ligament with a non-resorbable running suture and fixating it medially with interrupted absorbable sutures to protect the iliohypogastric nerve. Further refinements of the LR were advocated by Amid and David Chen, who minimized the skin incision (to approximately 5 cm), achieving comparable outcomes in the so-called ‘minimally invasive Lichtenstein’ technique and described “air-knots” medially in order to avoid damage to tiny nerve endings and prevent chronic pain. Importantly, in a modern Amid-modified LR, it is recommended that the surgeons actively assess for a possible femoral hernia—one of the limitations of LR—and, if present, repair it with a triangularly shaped mesh extension stitched to Cooper’s ligament [15].

**Figure 1:**
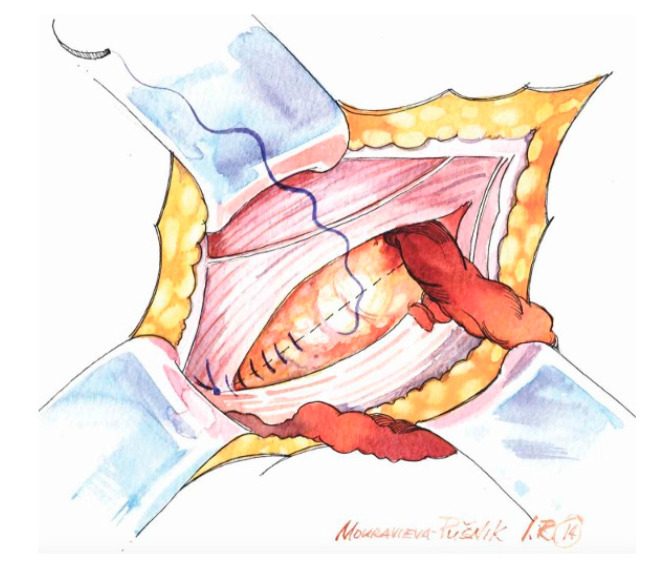
Suturing the inguinal floor with a running absorbable suture in a direct hernia.

**Figure 2:**
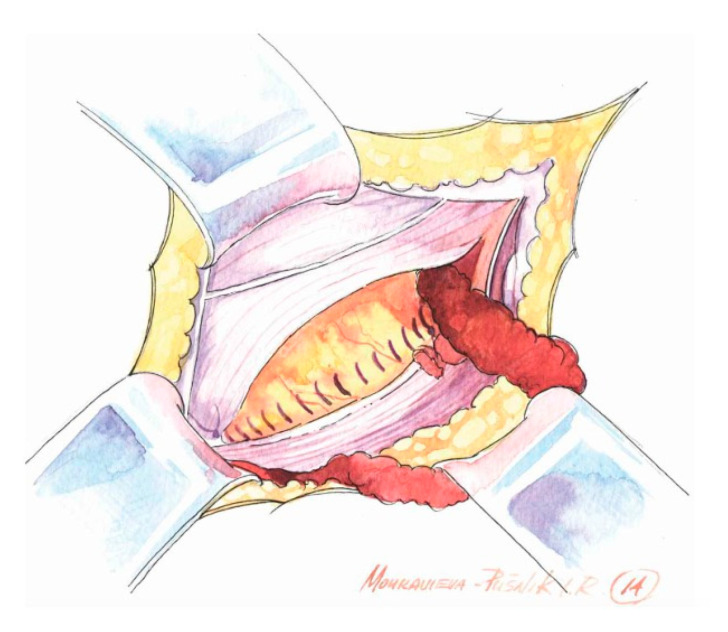
After completion of the suture, the inguinal floor is flat and the deep inguinal ring has shifted cranially.

**Figure 3:**
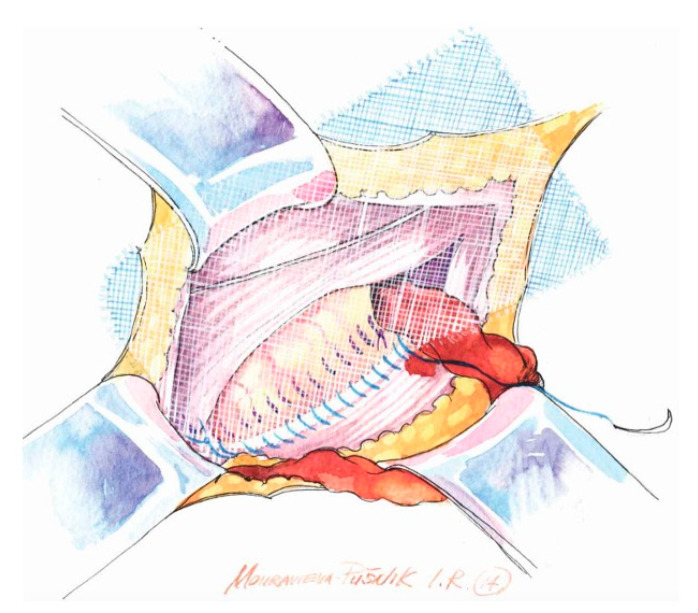
Large mesh is fixed with a running non-absorbable suture to the inguinal ligament. The overlap is at least 2 cm at the pubic tubercle.

**Figure 4:**
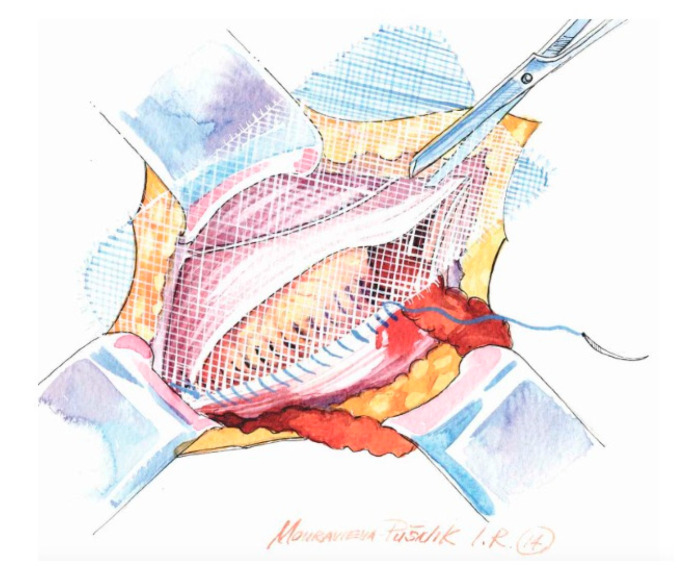
The suture ends just above the deep inguinal ring. The mesh tails are created individually according to the patient’s anatomy.

**Figure 5:**
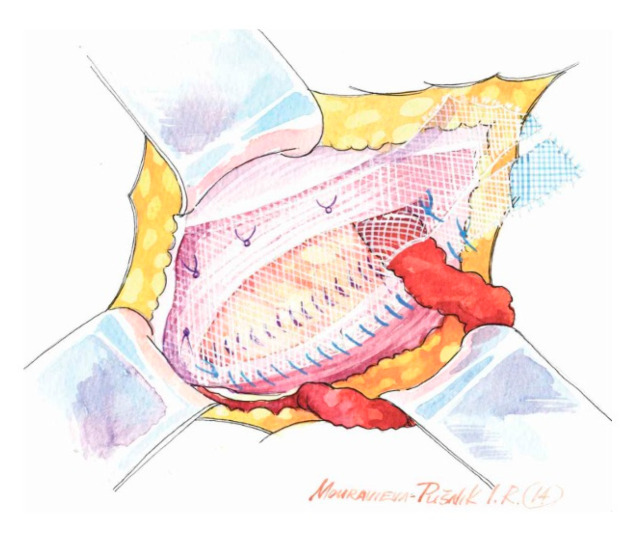
Fixation between two mesh tails and the inguinal ligament laterally and to the underlying internal oblique muscle medially.

**Figure 6:**
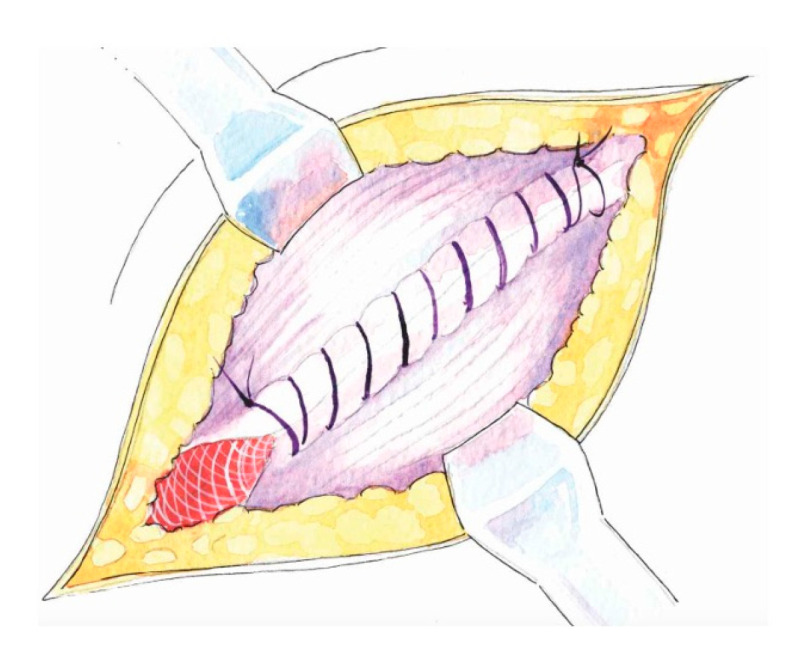
Closure of the external oblique aponeurosis.

Key principles of Lichtenstein Repair:

- Wide subaponeurotic dissection, including distal to the pubic tubercle, to allow for adequate mesh overlap;
- Identification and preservation of all three inguinal nerves (IIN, IHN, GbGFN) to avoid entrapment neuropathy, chronic pain, and unnecessary dysesthesias;
- Assessment of the femoral canal for a potential coexisting femoral hernia in every case;
- Preparation of the inguinal floor, with suture imbrication for direct hernias, and, in indirect hernias, Marcy-type suturing to cranially reposition the deep ring;
- Use of a sufficiently large mesh (≥12 × 8 cm) with ≥2 cm overlap beyond the pubic tubercle to avoid direct space recurrence;
- Continuous long-acting absorbable or non-resorbable suture to fixate the mesh to the inguinal ligament laterally (starting the suture at the firm fibrous tissue above the pubic tubercle) and interrupted absorbable “air-knot” sutures medially to protect the iliohypogastric nerve [5,6,15];
- Preservation of mesh tails with 4–5 cm of cephalad extension to account for coexisting interstitial or low-lying Spigelian hernias; overlapping the medial tail over the lateral with secure fixation of both tails to the inguinal ligament to create the mesh internal ring; consider fixation to Cooper’s ligament when femoral hernia is present.
- The key principles of Lichtenstein Repair are depicted in Figure 1, Figure 2, Figure 3, Figure 4, Figure 5 and Figure 6 below.

## 2. Methods

**Figure 7:**
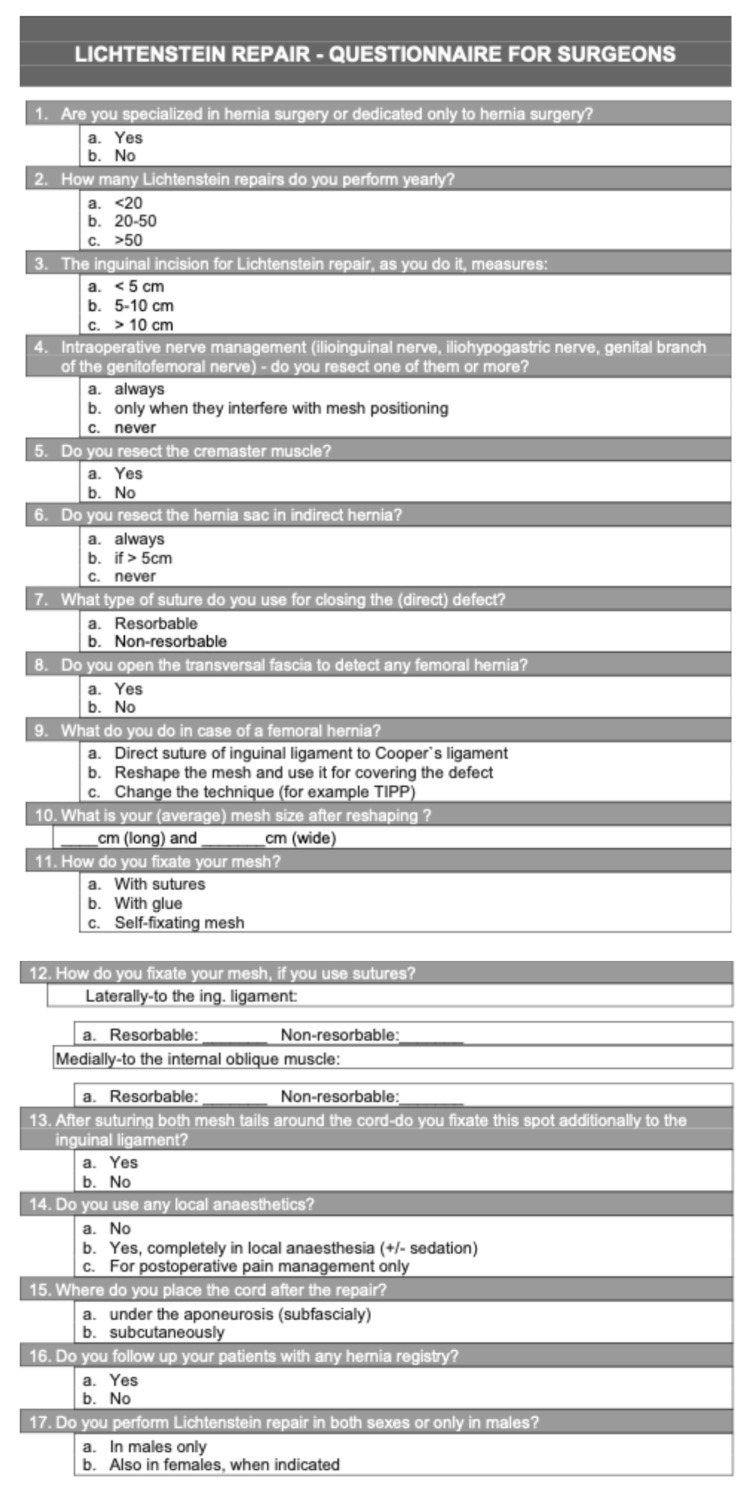
Questionnaire form.

In order to evaluate the reasons for variations in Lichtenstein Repair, a literature review of 7 major surgery textbooks was performed. Given that the key principles of the modern LR were described by the year 2004, only newer textbooks on LR have been examined. Additionally, to assess the reasons for intersurgeon LR variations, in the year 2023, a questionnaire with 17 questions concerning crucial steps of the LR was mailed to 90 surgeons who perform hernia surgery daily. Surgeons from public general hospitals in Carinthia, Austria (6 hospitals), and all public general hospitals in Slovenia (13 hospitals) were included. The response rate was 78% (70 surgeons). The questionnaire focused on key principles of the repair, as described by Lichtenstein and his successors. It was piloted in 2020, revised based on feedback, aligned with current guidelines on LR, and reviewed by external experts for content validity (Figure 7).

## 3. Results

Three classical surgical textbooks (*Bendavid’s Abdominal Wall Hernias Textbook* (2001) [16], *The Zollinger Atlas of Surgical Operations* (2016, 10th Edition) [17], and *Sabiston Textbook of Surgery* (2016, 20th Edition) [18] describe the LR in a similar way, which is summarized here.

An inguinal incision is made to expose the canal and the key anatomical structures, including the spermatic cord and inguinal ligament. The external oblique aponeurosis is opened, and the hernia sac—whether direct or indirect—is identified and reduced, preparing the posterior wall of the inguinal canal. A flat mesh is placed over the posterior wall, extending from the pubic tubercle to just beyond the internal ring, with its lower border on the inguinal ligament. The mesh is secured in a tension-free manner, with non-absorbable sutures to the inguinal ligament inferiorly and absorbable sutures medially, allowing the spermatic cord to lie comfortably, crossing the tails that are cut asymmetrically (1/3 laterally and 2/3 medially). The external oblique aponeurosis and subcutaneous tissue are then closed over the mesh, taking care to preserve nerves.

In Hope’s *Textbook of Hernia* (Springer 2017, 1st Edition) [19] and Schwartz’s *Principles of Surgery* (2019, 11th Edition) [20], all steps described above are included, including using a large mesh, checking for possible femoral hernia, and respect for skin nerves. Campanelli’s *The Art of Hernia Surgery* (Springer 2018, 1st Edition) [21] stresses the benefit of performing the LR under local anesthesia, mentioning personal modifications of possible and partial resection of the cremaster muscle. The crucial steps, like overlapping the pubic tubercle with the mesh, are underlined. On the contrary, Lorenz’s *Hernienschule Kompakt-Konkret-Komplex* (De Gruyter 2019, 1st Edition) [22] highlights the value of cremaster preservation, as described by Amid and Chen [15].

The questionnaire was completed by surgeons, performing LR at least once weekly (on average), with 86% being general surgeons without exclusive dedication to hernia surgery. We found that 68% of surgeons make short incisions and there is a tendency towards even smaller (<5 cm) skin incisions (21%). Sensory nerves are preserved absolutely by 25% of surgeons and resected only if necessary (by 67% of them). There was low (3%) intention to resect the nerves at all times. The cremasteric muscle was mostly preserved (94%), and indirect hernia sacs were only resected when bigger than 5 cm. We also found that 84% of surgeons prefer suturing the inguinal floor flat before mesh positioning. Only one third (32%) of surgeons would actively search for possible simultaneous femoral hernia. Of those who interrogate the femoral space, 73% intend to close the defect by a single suture to the Cooper’s ligament. Additionally, 75% of surgeons would choose a large mesh (15 × 9 cm or 12 × 7 cm). The majority of surgeons fixate the mesh with sutures in the recommended way (76%), and 77% of them fixate the sutured mesh tails to the inguinal ligament. A total of 84% of surgeons place the spermatic cord subfascially. Local anesthetic is mainly used as an adjunct in pain control (36%) rather than as the exclusive type of anesthesia (7%). Only 11% of surgeons use hernia registries to follow up their patients. LR would not be used as the primary option in females. Detailed results from the questionnaire are presented in Table 1.

## 4. Discussion

The present study offers several strengths. It compares LR, as described in seven current standard surgery textbooks. It draws on a multicenter sample that includes all public general hospitals in Slovenia and all public general hospitals in Carinthia (Austria), providing a comprehensive overview of current practice in these regions. The questionnaire specifically targeted the technically critical steps of Lichtenstein Repair—such as mesh size and configuration, exploration of the femoral canal, and nerve management—areas where small variations may have substantial clinical implications. Finally, the study evaluates daily surgical practices, enabling a direct comparison between evidence-based standards and real-world operative technique. Together, these elements allow the study not only to map variation but also to contextualize it within the evolving evidence base for anterior inguinal hernia repair.

The principles of Lichtenstein Repair (LR), although representing one of the most standardized and reproducible hernia techniques, are not uniformly or comprehensively described across major surgical textbooks. Bendavid’s *Abdominal Wall Hernias, The Zollinger Atlas of Surgical Operations,* and Sabiston’s *Textbook of Surgery* outline similar crucial steps but differ in emphasis and completeness. Bendavid mentions optional variations such as using a smaller skin incision in selected patients and omitting sac resection in smaller indirect hernias [16]. The *Zollinger Atlas* highlights specific operative nuances, including removal of cord lipomas and the use of interrupted absorbable sutures medially to reduce iliohypogastric nerve entrapment yet surprisingly promotes smaller mesh sizes without stressing the need for wide cephalad and medial overlap at the pubic tubercle [17]. Sabiston’s latest edition addresses these deficits by underscoring the importance of broad mesh overlap and correct fixation; however, its recommendation for a running medial suture is made without caution regarding chronic pain risk [18].

Newer textbooks provide a description more consistent with contemporary evidence-based LR. Hope’s *Textbook of Hernia* and Schwartz’s *Principles of Surgery* detail the modern technique, including the use of a short skin incision, careful cord handling, large mesh application, screening for femoral hernia, and nerve-respecting dissection [19,20]. Campanelli’s *The Art of Hernia Surgery* reinforces the essential components of LR and discusses the advantages of performing the operation under local anesthesia, while also presenting personal modifications such as partial cremasteric resection—an optional maneuver rather than a standard LR step [21]. Lorenz’s *Hernienschule* includes all LR-critical steps and introduces updated fixation options, such as glue or self-gripping meshes [22].

The variability in these descriptions demonstrates that surgical textbooks do not always provide a uniform, standardized, or fully evidence-based account of the LR technique. Only the most recent publications consistently reflect current consensus on key technical elements. Such inconsistency is concerning, as textbooks are expected to offer authoritative guidance for clinical decision-making and surgical training [23,24]. This highlights the need for more consistent and regularly updated educational resources to ensure accurate dissemination of LR best practices.

Most mesh-based inguinal hernia repair techniques show low recurrence rates, with Lichtenstein Repair demonstrating the most favorable outcomes [14]. Can the average (general) surgeon achieve these results? The current recurrence rate at 1-year follow-up after LR in the Herniamed registry for 350,959 patients (operated on mostly by non-specialized general surgeons) was 1.14%, which is a very favorable result [25]. It demonstrates that LR is easily reproducible even for surgeons who do not perform it on a weekly basis, once the crucial steps are respected [25,26]. Hernia experts have an even lower recurrence rate than non-expert hernia surgeons who operate on patients with inguinal hernias just occasionally [6,26].

In contrast to large registry datasets, which demonstrate low recurrence rates for LR across broad and heterogeneous populations, our multicenter sample provides a more granular, region-specific perspective on current practice in Slovenia and Carinthia. Our findings show that similarly favorable outcomes can be achieved in routine clinical settings across all public general hospitals, including those in which LR is not performed by high-volume hernia specialists. By documenting adherence to key operative steps and identifying areas of variation between institutions, our study extends prior registry analyses by demonstrating the reproducibility of LR under real-world conditions and highlighting specific practice patterns that may influence outcomes.

A larger skin incision is not a major problem for the final long-term outcome. It is well known that creases of the trunk are formed by well-organized collagen bundles in a beehive pattern, which enables greater skin mobility. The skin superior and inferior to the creases, including the inguinal crease, lacks the parallel organization of the collagen and elastic fibers, which are deposited in a random pattern [27]. Once the LR technique is mastered, it is no longer necessary to perform long skin incisions, unless the patient has increased inguinal adiposity or the hernia is large and/or scrotal. The so-called “minimal invasive Lichtenstein” with skin incisions smaller than 5 cm can easily be performed and is associated with reduced postoperative morbidity and improved pain control [15].

Precise knowledge of the nerve anatomy is crucial in decision-making for nerve-sparing preparation in the groin [28,29]. Although some authors report that prophylactic IIN-neurectomy in all patients significantly decreases the incidence of chronic groin pain after LR without added morbidities, recent recommendations do not support this statement [30]. Various surgical etiquettes have been proposed to reduce the incidence of unintentional intra-operative nerve injury [31]. Pragmatic neurectomy of the inguinal nerves, especially IIN, is recommended when nerve anatomy would interfere directly with the expected mesh position [32,33]. Other strategies, including elective resection of IIN, resulted in increased sensory loss at 6 and 12 months postoperatively [34]. In our questionnaire, the majority of surgeons (72%) performed pragmatic neurectomy of the IIN, while 25% of surgeons preserved all the nerves at all times; both strategies are considered good practice.

Preservation of the cremasteric muscle is widely supported in our questionnaire (94%), which is in accordance with studies and proper technique. Resection of the cremaster muscle seems reasonable in cases where excessive muscle tissue prevents adequate narrowing of the internal inguinal ring. In these cases, distal muscle stumps should be attached to the firm tissue around the pubic bone to prevent lowering of the testicle [6,30,31].

Hernia sac preservation (invagination), if the peritoneal sac is small, should not lead to higher recurrence rates and can prevent postoperative pain [35,36]. Some studies, however, including the Swedish registry with almost 50,000 patients, showed that sac invagination or resection without ligation might prevent short-term postoperative pain caused by ligation but may lead to a higher recurrence rate in indirect inguinal hernia, especially with large hernia sacs [37]. The results from our questionnaire show that only 12% of surgeons never resect the sac in indirect inguinal hernia. Almost two thirds of them (67%) resect the sac if it is larger than 5 cm, which is a reasonable solution according to European Hernia Society guidelines.

In larger M2- and M3-inguinal hernias, suturing of the inguinal floor is necessary in order to enable or optimize the mesh position. This can be achieved with interrupted stitches or a running resorbable suture. Recommendations warn against suturing into the periosteum of the pubic bone, which can cause severe postoperative pain and does not benefit the repair [38]. There are no clear recommendations for omitting (or using) this suture in indirect hernias. The resulting shift of the internal inguinal ring more cranially may reduce indirect recurrences, as with the Shouldice repair [39]. In our sample, the vast majority of surgeons (84%) suture the inguinal floor in order to flatten it prior to mesh positioning.

Although the incidence of femoral hernia in men is low (2%), it is necessary to check for its presence in LR and take appropriate action if found. Based on our questionnaire, awareness of this measure is low (32%). It is important to emphasize that both active screening for (possible) femoral hernias and repair of the defect in LR are part of this procedure [15]. In this case, most surgeons (73%) would opt for placing a non-absorbable suture between the inguinal ligament and Cooper’s ligament. LR also describes suturing the existing mesh patch to Cooper’s ligament as an option [15,40]. It is recognized that sutures placed on the inguinal ligament—particularly in tension-based repairs such as the Shouldice technique—combined with tissue retraction may widen the femoral ring and thereby increase the risk of developing a femoral hernia [39]. In contrast, a properly performed Lichtenstein Repair, as a tension-free technique, should not give rise to this potential complication.

Mesh size and positioning matter. Those that are too small without sufficient overlap can increase the recurrence rate [41]. There is no prescribed mesh size for LR. The recommended initial mesh size of 15 × 10 cm is sufficient for almost all patients, except those with very large scrotal hernias. The standard-sized mesh normally does not need to be shortened in length, but it can be shortened in width by 1–2 cm, in patients of diminished size, in whom it is trimmed according to the patient’s anatomy. A 15 × 8 cm mesh can generally be used for almost every LR, considering mesh shrinkage by 10% [42]. The key is in the generous preparation of the “landing zone” for the mesh. According to our questionnaire, 75% of surgeons would use a large mesh (15 × 9 cm) but almost a quarter of them would still use a mesh of 10 × 6 cm, which is considered too small. The use of undersized mesh in LR presumably plays an important role in recurrence [43].

The mesh material used in the hospitals where we sent the questionnaire was lightweight macroporous) polypropylene (pore diameter > 1 mm), according to guidelines [3,44]. Mesh fixation was analyzed in many studies and RCTs concerning recurrence, infection rate and chronic pain, but there were no significant differences noted [45,46] among many methods (sutures, tacks clips, adhesives, and self-gripping meshes). Studies agree that fixation of the mesh with a stitch to the firm tissue above the pubic tubercle and sufficient overlap are most important for preventing direct recurrence [3,6,15]. One study reported that self-gripping meshes may have more recurrences if the mesh is not additionally sutured to the inguinal ligament [47]. Atraumatic mesh fixation (glue) is suggested to reduce early postoperative pain [48]. According to the questionnaire, suturing of the mesh was performed adequately in 95% with a non-resorbable running suture to the inguinal ligament and in 92% with interrupted “loose-tie” stitches medially to the internal oblique. Overlapping (medial tail over lateral tail) and suturing of the mesh tails to the inguinal ligament represent an important step in LR. The majority (77%) of surgeons recognize this as a step that lowers the chance of indirect recurrence.

We found that 57% of surgeons do not perform LR under local anesthesia. This is due to differences in individual experience, local conditions in individual hospitals, and, last but not least, the comfort of surgeons in performing the procedure [49]. The use of local anesthetics as the main method of anesthesia could optimally be more widespread in the performance of LR.

The postoperative position of the cord was subaponeurotic in 84% (in contact with the mesh), which offers additional protection to the cord structures compared to cord position in the subcutaneous tissue (16%). Cord contact with the mesh should not cause obstruction of the vas deferens as the cremasteric is not stripped in the modern LR [50].

LR should not be a method of choice for primary inguinal hernia in women [51]. Accordingly, most surgeons (94%) in the questionnaire did not opt for LR in women, except for exceptional cases where the transperitoneal approach (TAPP, TIPP) was not possible or reasonable.

Surgeons participating in a registry report better effectiveness than non-participants and complement the results of RCTs [52]. They provide long-term monitoring of surgical quality and facilitate surgical care improvements at individual facilities [53]. It is thus concerning that the questionnaire showed that only a small minority (10%) of surgeons participate in registries or follow the results of their patients systematically. This directly limits adequate evaluation of any of the above-mentioned considerations of the LR. Hernia registries enable the estimation of exact recurrence rates, chronic pain incidence, and other complications [54].

The variations, according to the questionnaire (e.g., undersized mesh, limited femoral exploration, lack of registry follow-up) hypothesize higher recurrence or chronic pain, yet it is important to underline that no patient-level outcomes were collected with our survey. Such associations should therefore be presented as hypotheses or literature-based extrapolations rather than conclusions. In our study, these variations were simply unexpected deviations from current guidelines.

Tension-free mesh open repair for inguinal hernia has a long history. The modern Amid-modified LR is considered to be superior to other open techniques [55]. Many evidence-based modifications arose after the originally described operation by Irving Lichtenstein and were incorporated into the modern technique. Any novel modifications to the LR technique should not interfere with the established key principles of LR without careful scientific deliberation.

### Future Directions

Future research should evaluate how variations in key principles of LR influence recurrence and chronic pain. Increased registry participation and standardized training could help reduce practice variability and improve outcomes across general surgical settings.

## 5. Conclusions

With Lichtenstein Repair, the pioneering era of open inguinal hernia repair is over. Despite LR’s continuous evolution, individual modifications of the LR technique should not interfere with the key principles, as described by the authors of the Lichtenstein Amid Hernia Clinic, in order to obtain optimal results. Teaching the original Amid-modified LR to young surgeons can improve the bias of comparing different techniques under the cover name of LR in the future.

## Figures and Tables

**Table 1:** Survey results for technical variations in Lichtenstein Repair according to questionnaire; *n* = total number of surgeons involved.

Question According to Questionnaire	Answer	No. of Surgeons (Total 70)	%
**Caseload of LR per surgeon/year**	<30	*n* = 19	27%
	30–50	*n* = 37	53%
	>50	*n* = 14	20%
**Hernia sub-specialization**	Yes	*n* = 10	14%
	No	*n* = 60	86%
**Skin incision size**	<5 cm	*n* = 15	21%
	5–10 cm	*n* = 47	68%
	>10 cm	*n* = 8	11%
**Surgical management of sensory nerves**	Never resect	*n* = 18	25%
	Resect when needed	*n* = 50	72%
	Always resect	*n* = 2	3%
**Cremaster muscle resection**	Yes, always	*n* = 1	1%
	Yes, if necessary	*n* = 4	5%
	No	*n* = 65	94%
**Hernia sac resection** **(indirect)**	Always resect	*n* = 8	11%
	Resect when >5 cm	*n* = 53	77%
	Never resect	*n* = 9	12%
**Sutures of the inguinal floor**	Yes	*n* = 59	84%
	No	*n* = 11	16%
**Checking for femoral hernia**	Yes	*n* = 22	32%
	No	*n* = 48	68%
**Synchronous femoral repair**	Suture to Cooper’s ligament	*n* = 51	73%
	Reshape mesh and cover defect	*n* = 13	19%
	Switch to TIPP technique	*n* = 6	8%
**Final mesh size after shaping**	15 × 10 cm	*n* = 30	43%
	12 × 7 cm	*n* = 22	32%
	10 × 6 cm	*n* = 18	25%
**How to fixate the mesh**	Sutures	*n* = 53	76%
	Glue	*n* = 9	13%
	Self-gripping mesh	*n* = 8	11%
**Suture fixation**	Laterally absorbable	*n* = 4	5%
	Laterally non-absorbable	*n* = 66	95%
	Medially absorbable	*n* = 64	92%
	Medially non-absorbable	*n* = 6	8%
**Suture of the mesh tails**	Yes	*n* = 54	77%
	No	*n* = 16	23%
**The role of local anesthesia (LA)**	None	*n* = 40	57%
	Op. in LA only	*n* = 5	7%
	For postop. pain control	*n* = 25	36%
**Spermatic cord position**	Subaponeurotically	*n* = 59	84%
	Subcutaneously	*n* = 11	16%
**Cooperation with hernia registry**	Yes	*n* = 8	11%
	No	*n* = 62	89%
**Use of Lichtenstein Repair in females**	In males only	*n* = 66	94%
	Also in females (when indicated)	*n* = 4	6%

## Data Availability

The data supporting the findings of this study are not publicly available.
